# Challenges in the Management of Invasive Fungal Infections in the Middle East: Expert Opinion to Optimize Management Using a Multidisciplinary Approach

**DOI:** 10.7759/cureus.44356

**Published:** 2023-08-30

**Authors:** Reem S AlMaghrabi, Tariq Al-Musawi, Osama Albaksami, Ahmad L Subhi, Riad E Fakih, Neil R Stone

**Affiliations:** 1 Department of Medicine, Organ Transplant Center, King Faisal Specialist Hospital and Research Centre, Riyadh, SAU; 2 Department of Critical Care Medicine, Al Salam Hospital, Al-Khobar, SAU; 3 Department of Medicine, Royal College of Surgeons in Ireland - Bahrain, Busaiteen, BHR; 4 Department of Infectious Diseases, Infectious Disease Hospital, Kuwait City, KWT; 5 Department of Infectious Diseases, Al-Qassimi Hospital, Sharjah, ARE; 6 Department of Hematology, King Faisal Specialist Hospital and Research Centre, Riyadh, SAU; 7 Department of Clinical Research, Alfaisal University, Riyadh, SAU; 8 Department of Microbiology, Hospital for Tropical Diseases, London, GBR; 9 Department of Microbiology, University College London Hospitals, London, GBR

**Keywords:** multidisciplinary team, middle east, invasive fungal infections, epidemiology, antifungal treatment

## Abstract

Invasive fungal infection (IFI) is a significant global healthcare concern among critically ill and immunocompromised patients. In Middle Eastern countries, IFI has been steadily increasing among hospitalized patients in the past two decades. Diagnosis of IFI at an early stage is crucial for efficient management. Invasive fungal infection management is complex and requires the involvement of physicians from different specialties.

There are several challenges associated with IFI management in the countries in the Middle East. This review aims to understand the key challenges associated with IFI management in the Middle East, encompassing epidemiology, diagnosis, therapeutic options, and optimizing a multidisciplinary approach. In addition, this review aims to incorporate expert opinions from multidisciplinary fields for optimizing IFI management in different Middle Eastern countries by addressing key decision points throughout the patient’s journey. Lack of epidemiological data on fungal infections, slow and poorly sensitive conventional culture-based diagnostic tests, limited availability of biomarker testing, lack of awareness of clinical symptoms of the disease, limited knowledge on fungal infections, lack of local practice guidelines, and complicated disease management are the major challenges associated with IFI diagnosis and management in the Middle Eastern countries. Implementation of a multidisciplinary approach, antifungal stewardship, improved knowledge of fungal infections, the use of rapid diagnostic tests, and enhanced epidemiological research are warranted to lower the IFI burden in the Middle East.

## Introduction and background

Invasive fungal infections (IFIs) are an important global healthcare concern for immunocompromised and critically ill patients [1]. Fungal pathogens are responsible for at least 13 million infections and more than 1.5 million deaths annually worldwide, mostly in immunocompromised patients [2]. Moreover, about 1.9 million people globally are estimated to develop an acute IFI annually [3]. In the Middle East, the incidence of IFI in hospitalized patients has steadily increased over the past two decades. However, the incidence of specific IFIs varies from one report to another [4,5]. For example, the overall incidence of invasive candidiasis (IC) ranged from 0.15 cases per 1,000 hospital discharges in Kuwait to 1.55-1.65 cases per 1,000 hospital discharges in Saudi Arabia [5]. Major risk factors for IFI include the use of broad-spectrum antibiotics, critical illness, invasive medical devices, cytotoxic chemo-intensive therapy, steroid exposure, immunosuppressive therapy, and hematopoietic or solid organ transplantation [6-8]. One major concern about IFI is the diagnostic challenge because of nonspecific and non-localizing clinical presentations and variability owing to the host’s immunity and physiological conditions [7,9]. Another significant challenge is the lack of sensitivity of the diagnostic methods; for example, biomarkers lack sensitivity and specificity, and even modern techniques such as polymerase chain reaction (PCR) are unable to distinguish colonization from infection [9-11]. Moreover, the involvement of mycologists in IFI management is crucial; however, there is a lack of experts in this field [12].

Invasive fungal infections are known to cause significant morbidity and mortality [4]. Early diagnosis and effective treatment initiation are essential. Because of poor diagnostics, non-specific clinical presentation, high mortality in untreated infections, the cost-ineffectiveness and toxicity of antifungals, and the need for prolonged therapy, the IFI management approach is highly complex. For effective management, it is essential to implement a multidisciplinary approach that involves specialists from various disciplines who can guide management on individual cases, develop local diagnostic and treatment guidelines for fungal infections, and identify healthcare and research needs in the field of fungal infections at the local, regional, and international level. There is currently a paucity of data related to IFI epidemiology, treatment guidelines, mortality, and a multidisciplinary approach to managing IFIs in Middle Eastern countries [4].

This review aims to understand the key challenges associated with IFI management in the Middle East, encompassing epidemiology, diagnosis, therapeutic options, optimizing a multidisciplinary approach, and incorporating the opinions of experts from multidisciplinary fields to overcome these challenges.

## Review

Challenges associated with IFI management

Epidemiological Challenges

Globally, there has been a change in the epidemiology of IFI over time, owing to the rise in at-risk populations (immunosuppression, rising incidence of diabetes) [8]. Environmental factors, patient characteristics, and exposure to antifungal agents are all anticipated to increasingly affect IFI epidemiology [4,8]. Knowledge of the local epidemiology of fungal infections in a country or region is important to provide optimal management in terms of drug interventions, infection prevention, and control. Although information on epidemiology and the burden of fungal infections is available for Western Europe and North America [2,13,14], limited information is available for the Middle East [4]. Table 1 depicts the epidemiological burden of IFI in the Middle East.

**Table 1:** The epidemiological burden of different IFIs in Middle Eastern countries

Serious fungal infections	Incidence rates/100,000 patients				
	Qatar [15]	Oman [16]	Kuwait [17]	Jordan [18]	Saudi Arabia [18]
Cryptococcal meningitis	0.43	0.02	0	0	—
*Pneumocystis* pneumonia	0.8	0.11	0.1	0.1	—
Oral candidiasis	6.52	237	1.7	0.14	—
Esophageal candidiasis		1.5	0.8	1.0	1.4
Invasive aspergillosis	0.60	5.4	16.7	1.34	7.6
Chronic pulmonary aspergillosis – all	26.82	3.4	21.3	11	3.4
Allergic bronchopulmonary aspergillosis (ABPA)	60.2	141	187	141	212
Severe asthma with fungal sensitization (SAFS)	79.46	85	246	186	280
Candidemia+	15.4	5.0	6.8	5.0	10
*Candida* peritonitis	8.02	0.75	3.5	0.75	1.6
Recurrent *Candida* vaginitis (≥4 × per year)	3506	2446	2595	3097	33.20
Mucormycosis	1.23	0.2	0.5	0.02	0.034
Fungal keratitis	0.32	12	15.5	—	—

Because of the limited number of studies, many aspects of IFIs, such as clinical characteristics, prognostic factors, infecting species, and antifungal susceptibility, remain poorly understood.

Invasive candidiasis (IC), particularly candidemia, is the most studied IFI in the Middle East. Research from different Middle Eastern countries (Saudi Arabia, Qatar, Kuwait, Jordan, United Arab Emirates (UAE), Bahrain) found *C. albicans* to be the most frequently isolated (22.3%-60%) *Candida* species, in addition to *C*. *auris*, *C*. *tropicalis*, *C*. *parapsilosis*, and *C*. *glabrata* from blood cultures for candidemia [19,20]. This is similar in distribution to other regions of the world. However, the epidemiology of candidemia and IC in different patient populations (e.g., intensive care unit (ICU), neutropenic) has not been studied extensively in the Middle East [19,21]. There is also a lack of published regional studies specifically on invasive aspergillosis (IA), other invasive mold infections, and infection with dimorphic fungi such as histoplasmosis in this region [5].

Antifungal Resistance

The development of resistance to antifungal drugs is a rising global concern. Invasive *Candida* infections are reported to be significantly resistant to antifungal therapy (AFT) [22]. In Kuwait, candidiasis has shown a rapid emergence of antifungal resistance with a high associated mortality rate of 47% [23]. There have also been reports of increased fluconazole resistance in different *Candida *species, such as *C*. *parapsilosis* and *C*. *glabrata* [22,24]. The greatest threat, however, is *C*. *auris*, a multi-drug-resistant *Candida* species (emerged in 2009 in Japan) responsible for severe hospital outbreaks in the Middle Eastern region [25,26]. Elevated rates of antifungal resistance with decreased sensitivity to azoles, polyenes, and echinocandins were reported in *C*. *auris* [27]. A Qatar-based study on *C*. *auris* isolates has reported 70% resistance to fluconazole and amphotericin B [28]. In 2022, *C*. *auris* has been named in the 'critical' category of the WHO list of priority fungal pathogens [29]. Furthermore, reports from Kuwait suggest azole-resistance (mainly itraconazole) rates as high as 7% and 12.5% in *A*. *fumigatus* environmental and clinical isolates, respectively [30,31]. However, detailed information on rates and risk factors for antifungal resistance in the Middle East is not available.

Diagnostic Challenges

Limitations of fungal diagnostic tests: Diagnostic tests for IFIs include histopathologic examination, radiologic evidence, conventional mycologic methods (such as culture and susceptibility, serologic methods), serum biomarkers, and molecular techniques based on PCR [9,14]. Direct microscopic examination and culturing of clinical samples (tissue, sputum, urine, or blood) to isolate the etiological fungal agent is considered the gold standard for IFI diagnosis [9,11]. However, these traditional diagnostic techniques are not efficacious due to the slow turnaround time and invasive nature of the specimens required for testing. Moreover, it is not feasible to wait several days for fungal culture results in patients who are in critical care or undergoing chemotherapy and may require immediate antifungal treatment (AFT). Early AFT is presumed to reduce mortality in high-risk patients; however, there is very limited evidence to demonstrate mortality benefits following AFT [32]. Hence, prompt decision-making and appropriate AFT initiation are required for this type of patient. In some settings, biomarker assays or nucleic acid amplification tests (NAATs) on blood, bronchoalveolar lavage (BAL) fluid, or urine samples may lead to an earlier confirmation of a diagnosis of IFI. However, such diagnostic modalities are limited, and there is an incomplete consensus on their performance standards and interpretive criteria across different settings and patient populations [33,34]. Rapid tests such as point-of-care galactomannan (GM) assays or histoplasma antigen detection are increasingly becoming available and are of interest to critically ill patients who require a rapid turnaround of results [11].

Limited availability of biomarkers: Due to the limitations of conventional culture-based diagnostic tests for invasive mycoses, there is an increasing use of non-culture-based techniques, such as biomarker tests for IFI diagnosis [35]. A 1-3 β-D-glucan (BDG) test for detecting 1-3 BDG (fungal cell wall component) in the serum of an infected individual may assist in a rapid diagnosis of certain IFIs, including candidiasis, aspergillosis, and *Pneumocystis* pneumonia (PCP) [34]. Galactomannan (GM), a cell wall component of some pathogenic molds, is used to detect invasive mycoses infections, particularly *Aspergillus* infection [33,34,36]. Although these tests are performed on easy-to-acquire serum samples with a relatively short turnaround time, they may not be readily available, especially in low-resource settings [37]. Further, their appropriate clinical interpretation requires expertise that may not always be accessible [33,34,36]. Moreover, there is a great disparity in access to fungal biomarkers such as BDG and GM internationally and within Middle Eastern regions. Many centers across the Middle East have no ‘in-house’ access to these tests, requiring sending away samples to a reference laboratory, which in turn causes an unacceptable delay in getting the result, rendering it clinically useless [38].

Limited accessibility to molecular testing: Molecular diagnostic techniques, mainly NAAT and PCR, are increasingly used in IFI diagnosis. Over the years, real-time PCR methods for *Aspergillus* detection mainly use ribosomal ribonucleic acid (rRNA) genes for amplification with several different options, such as 18S ribosomal deoxyribonucleic acid (rDNA), the 28S rRNA, the 5.8S rDNA, and also internal transcribed spacer (ITS) regions between these genes [11,39]. Previously, the European Organization for Research and Treatment of Cancer and the Mycoses Study Group Education and Research Consortium (EORTC/MSGERC) consensus in 2008 excluded both NAAT and PCR as prominent diagnostic techniques due to a lack of standardization and validation [40]. However, revised guidelines favor the real-time PCR of samples (such as blood, serum, BAL fluid, or expectorated sputum) for the detection of the majority of IFIs [40]. Despite this, molecular diagnostics is an issue, as it remains unavailable beyond a few specialist centers. Besides aspergillosis, 18S rDNA and ITS PCR are increasingly used as a ‘pan fungal’ PCR for the diagnosis of a wide range of suspected fungal species, including mucormycosis, *Pneumocystis* *jirovecii* pneumonia, *Candida* infections, and some endemic mycoses such as coccidioidomycosis [11]. Access to diagnostics is inconsistent, and some centers in Middle Eastern countries even send samples to Western European countries for IFI confirmation [19,41]. This increases costs and turnaround time, which negatively impacts treatment. Local access to the best available diagnostics for fungal infections, as well as expertise from specialists in fungal disease, are ideal for good outcomes, which are not yet reached in much of the region. 

Management Challenges

Limited knowledge of mycology and fungal infections: The management of patients with IFI poses a challenge to healthcare professionals (HCPs) due to their limited awareness of the symptoms, diagnosis, and use of antifungal agents. Mycology is a smaller niche within medicine and is often poorly covered in medical school curricula, leading to a lack of expertise and knowledge even among infectious disease (ID) as well as respiratory specialists [12]. Historically, there has been a lack of attention to fungal infections like aspergillosis and other infections in terms of education, funding, and research [12]. Moreover, the treatment of immunocompromised patients with IFI is particularly complex. This is because antifungals have significant side effects, and their inappropriate use might expose patients to undue toxicity and interactions with other drugs used to treat them [42]. For example, azole-group drugs such as voriconazole exhibit complex pharmacokinetics and significant drug-drug interactions (DDI) with several classes of co-administered drugs [43]. Furthermore, due to the lack of appropriate diagnosis as well as knowledge of DDIs and antifungal resistance, there is an increased use of empiric treatment in high-risk group patients instead of targeted therapy [41, 44]. Often, empirical treatment approaches result in antifungal administration without any radiological or microbiological evidence [45]. Hence, effective management of IFI, already difficult due to poor diagnostics, is further limited by a lack of expertise, resources, and awareness within disciplines across the healthcare community [42].

Lack of healthcare records in IFI management: There is a lack of robust epidemiological data and healthcare information for necessary stakeholders such as HCPs and public healthcare policymakers. While electronic health records are used in most Middle Eastern countries, updating the database to include information on laboratory indicators and drug usage to optimize IFI management is limited. Recently, a data acquisition model called Optum® has been implemented in the US to collate unidentified data on invasive mucormycosis [46]. The development of such prediction models can be useful in the Middle East region to collate data on diverse types of IFIs, including causal pathogens, AFT used, and patient outcomes. A unified registry of cases would allow for descriptive epidemiology, informed public health and patient care treatment, and policy decisions around antifungal use and diagnosis of IFI.

Challenges Associated with Therapeutic Options and Guidelines

Resistance to antifungal drugs: One of the primary challenges in IFI management in the Middle East is the lack of local or regional treatment guidelines for IC and IA. International guidelines such as those of the Infectious Diseases Society of America (IDSA) and the American Society of Clinical Oncology (ASCO) are primarily followed by physicians in this region for the management of both IC and IA [47]. For invasive *Candida* infections, both IDSA and experts from the Middle East primarily recommended echinocandin (micafungin, anidulafungin, and caspofungin) for patients with prior azole exposure [47]. A study conducted on patients in Saudi Arabia with *Candida* infection showed a higher likelihood of developing antifungal resistance in those with previous echinocandin exposure [22]. In contrast, another Saudi Arabia-based study examining different *Candida* species (*C*. *albicans*, *C*. *glabrata*, *C*. *parapsilosis*, and *C*. *tropicalis*) showed 100% susceptibility toward echinocandins compared to 41.5% for fluconazole [48]. This highlights the need for local epidemiology to develop local guidelines. Moreover, the resistance patterns of different fungal species toward various antifungal agents are not well understood. There is also a lack of robust data on drug resistance in different countries in this region, which poses a challenge in optimizing IFI treatment management relevant to the region. The development of local guidelines by the experts within the region is essential to guide clinicians in a way that is relevant epidemiologically and realistically in terms of access to diagnostic tests and antifungal drugs.

Therapeutic drug monitoring: It is important to have knowledge of the pharmacokinetic and pharmacodynamic behavior of available antifungal agents. Therapeutic drug monitoring (TDM) is now regarded as an important tool to enhance the effectiveness of antifungals and reduce their toxicity. Therapeutic drug monitoring of all types of drugs cannot be performed in all hospitals, particularly those drugs that need a rapid response and depend on analytical techniques not readily available in traditional clinical laboratories. Primarily, triazoles and flucytosine are recommended for routine TDM [41,49]. However, in the Middle East, only 53% of clinicians routinely use TDM for the right indication and correct sampling time [50]. Increased awareness of the need for TDM and improved access to assays to measure drug levels are required in the region.

Accessibility to antifungal drugs: The initiation of AFT depends on several factors, such as activity, dosing, safety profiles, costs, underlying disease conditions, and surgical complications. Besides, accessibility and affordability of medications are major barriers to improved outcomes in lower- and middle-income countries. For example, the lack of availability of flucytosine, used mainly to treat *Cryptococcus *infection, makes it inaccessible in many low-to-middle-income countries [51]. The cost of drugs remains another major barrier. One of the newer azoles, isavuconazole, used to treat aspergillosis and as an alternative therapy for mucormycosis, was approved in the US in 2015, whereas it recently became accessible in the Middle Eastern countries but remains prohibitive for some centers due to its high cost.

Multidisciplinary Approach

**Figure 1:**
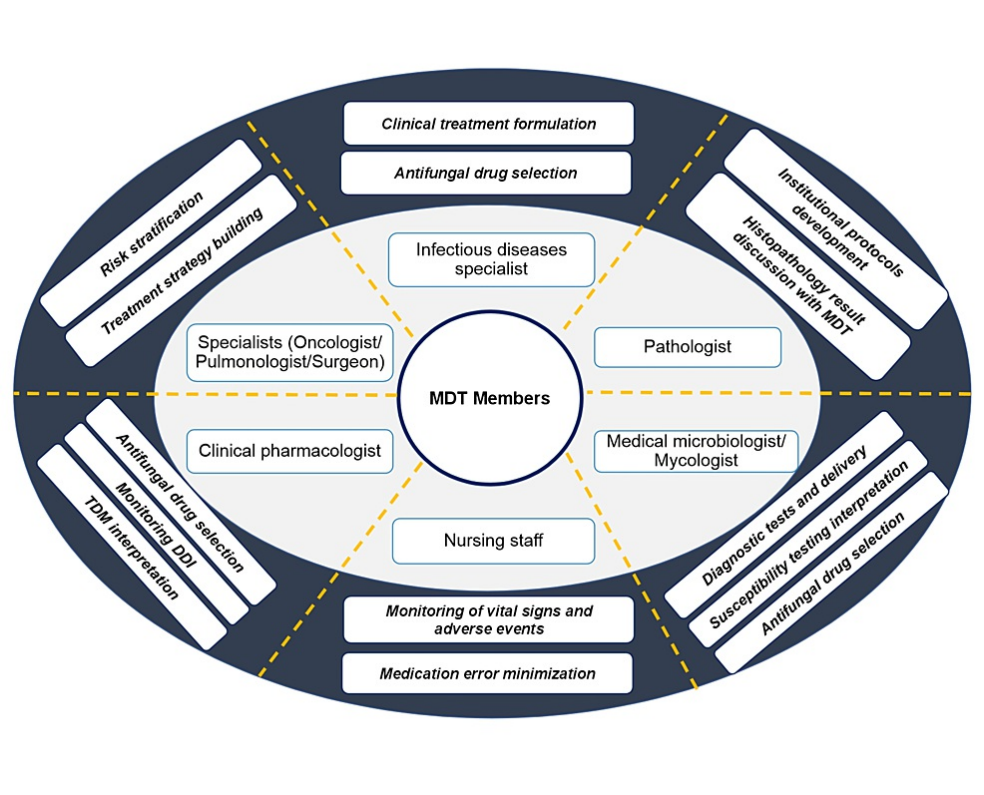
An MDT Approach in IFI management Adapted from [50] DDI: drug-drug interactions; IFI: invasive fungal infection; MDT: multidisciplinary approach; TDM: therapeutic drug monitoring

Invasive fungal infection affects high-risk group patients (immunocompromised patients, critically ill patients, transplant recipients); thus, treating physicians need to be aware of the ever-growing range and risk of fungal pathogens, apply and interpret new diagnostic approaches, and select appropriate antifungal agents for optimal management of infection [52]. In addition, they should be aware of different DDIs that are often a significant problem in critically ill patients or patients undergoing chemotherapy, who are often on polypharmacy [53]. Due to the complex nature of diagnosis and managing IFI, it is essential to have a multidisciplinary team (MDT) of HCPs such as general physicians, pathologists, mycologists, ICU specialists, ID specialists, hemato-oncologists, nurses, and pharmacists, together with specialist knowledge and experience from a wide variety of backgrounds (Figure 1) [52,54].

It is vital that members of MDT have a common treatment goal and reach a consensus on the timely treatment initiation, duration, escalation, and de-escalation of treatment to obtain positive outcomes [52]. In the MDT approach, multidisciplinary rounds of assessment are conducted to evaluate patients’ conditions. These rounds help in embracing evidence-based practices and also confirm effective communication among caregivers [55]. Implementation of an MDT protocol has significantly improved patient outcomes, including length of ICU stay reduction and a lower mortality rate for critical patients [55].

In Middle Eastern countries, most clinicians follow international guidelines for IFI treatment in the absence of local or regional guidance [47]. The adoption of these guidelines at the institutional level might have a few logistic challenges, such as a lack of coordination and cooperation among different specialists who are an integral part of caring for patients with IFI. To overcome these challenges, the IFI-MDT model has been proposed to promote and provide sufficient care and treatment to patients with IFI [52]. There is a limited report on the implementation of MDTs in the Middle East. Recently, a study from the healthcare system of Saudi Arabia reported on the use of MDT in IFI management [55].

Expert opinions to optimize IFI management in the Middle East

Based on the challenges described in this review, we propose the following steps required to improve the management of IFI in the Middle East:

Conducting Epidemiological Research and Developing a Nationwide Fungal Registry

**Figure 2:**
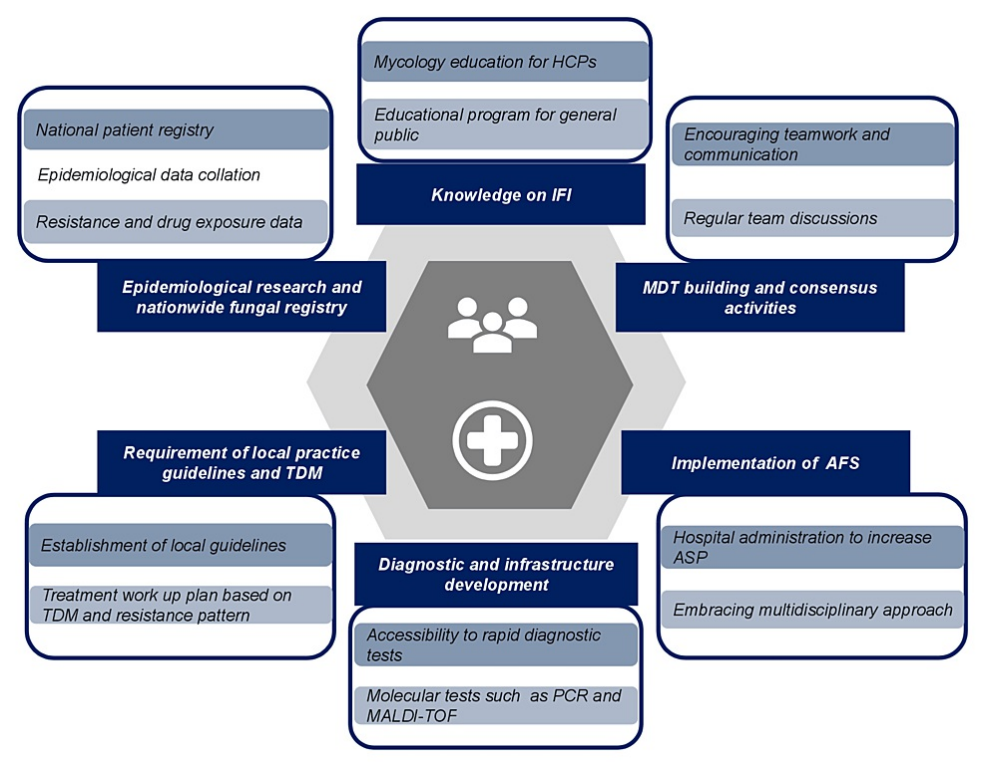
Expert recommendations for IFI management in the Middle East Image created by the authors. AFS: antifungal stewardship; ASP: antifungal stewardship program; HCPs: healthcare professionals; IFI: invasive fungal infections; MALDI-TOF: matrix-assisted laser desorption/ionization time of flight; PCR: polymerase chain reaction; TDM: therapeutic drug monitoring

A clear understanding of the regional epidemiological picture of IFI is essential. This can be accomplished by establishing a national surveillance and database system in the Middle East. FungiScope®, a global registry, was developed to focus on epidemiology, pathogen biology, and the clinical course of IFI to improve knowledge of epidemiology and subsequently improve patient management [56]. Similarly, the creation of a region-based registry can give a more accurate understanding of the scope and impact of IFI and region-based epidemiology and develop strategic healthcare provision planning (Figure 2).

Requirement of Local Practice Guidelines and TDM

The establishment of local practice guidelines for IFI management is essential in Middle Eastern countries to provide optimal treatment for patients suffering from invasive mycoses [41]. Since IDSA and ASCO-based guidelines are primarily practiced in clinical settings in North America and Europe, policymakers can focus on regional epidemiology to understand the most appropriate antifungal prophylaxis and treatments for the Middle Eastern population. Local guidelines can also include appropriate dosing and duration regimens for antifungals in the local setting.

Therapeutic drug monitoring can be implemented to assess whether a patient is not responsive to a particular class of antifungals. The fundamental goal of TDM is to personalize the dosage to achieve the highest level of therapeutic efficacy and minimize drug-related adverse effects. Partially available in Saudi Arabia and Kuwait, TDM services can provide an excellent pharmacological response with fewer adverse toxic drug effects [57]. Many hospitals affiliated with the Ministry of Health in Saudi Arabia have recently started offering TDM services as a regular aspect of patient management and drug monitoring [57]. More such collaborations are warranted to enhance TDM activities in these countries and to make TDM available across the region (Figure 2).

Diagnostic and Infrastructure Development

Increasing awareness among healthcare practitioners can help identify the infection at an early stage. This can be particularly helpful for critical care patients and immunosuppressed patients who may require immediate treatment. Further, access to biomarker testing facilities should also be improved. Reference laboratories working as primary testing centers for a group of laboratories in specific regions of each country can help with fast-tracking diagnostic tests for proper supply and logistics [3].

Implementation of MDT Protocols

The MDT approach is a collaborative approach to bring HCPs of diverse specialties together and agree upon the best treatment workup plan for patients with IFI [52] (Figure 1). The unified goal of better patient outcomes remains central to all members of the MDT, regardless of their specialty expertise, and this common goal forms the basis of effective MDT work [52]. Adoption of an agreed MDT protocol in Middle Eastern countries is essential to improving the management of patients with suspected IFI. A consensus MDT policy can encourage caregivers to cooperate and share relevant clinical information important for patient care, thereby facilitating the clinical decision-making process.

The MDT model is dependent on the interpersonal skills of the team members. With a focus on enhanced and effective communication and collaboration, consensus activities within the MDT through regular discussions can optimize the treatment process (Figure 2). For better collaboration, MDT members should also be well-versed in the process and outcome metrics, regional and national trends in fungal infection rates in high-risk categories, and the AFS [52]. In addition, a supportive environment for teamwork must be provided by the hospital administrators, and for this, the hospitals should have adequate multidisciplinary staff to take care of the patient’s requirements.

Implementation of Antifungal Stewardship

Effective management of IFI depends on the initiation of appropriate AFT that needs to be optimized in terms of cost, availability, affordability, and toxicity. Globally, numerous institutions have adopted a multidisciplinary AFS approach to optimize the use of antifungals, reduce antifungal resistance, and improve patient outcomes, and this depends on a timely and accurate diagnosis of IFS and the causative agent [58]. The use of the AFS program has demonstrated a significant decrease in antifungal consumption, time to targeted therapy and length of empirical therapy, cost burden, length of hospital stays, and mortality rate [58]. A UAE-based study revealed that the involvement of MDT resulted in the improved implementation of an antifungal stewardship program (ASP) among ICU patients [59]. Considering the benefits of AFS, it becomes imperative to implement this in the Middle East due to the numerous outbreaks of *C. auris* in various Middle Eastern countries such as Kuwait, Qatar, Saudi Arabia, the UAE, Iran, Sudan, Lebanon, and Oman [60-62]. 

Developing Awareness of IFI Through Education

Despite the alarming impact of fungal diseases on human health, fungal infections still do not receive the same importance as bacterial or viral infections, whether in medical school curricula or research funding. A lack of basic understanding of IFI awareness among physicians was reported in a study [63]. This lack of expertise can lead to a delayed diagnosis of IFI because of a lack of clinical suspicion, thereby resulting in a delay in ordering and correctly interpreting diagnostic tests for IFI. The diagnosis of fungal infection is further complicated by a lack of sufficient knowledge in the field of laboratory and clinical mycology. Mycology laboratory specialists are essential to diagnosing and identifying the ever-changing landscape of fungal pathogens and their antifungal susceptibility. However, there remains a paucity of adequately trained mycologists [64]. Thus, advanced medical mycology education is essential for clinicians and laboratory specialists. Middle Eastern countries can conduct collaborative multidisciplinary mycological meetings, similar to other regional groupings such as the Asia Fungal Working Group or the Pan African Mycology Working Group (under the International Society for Human and Animal Mycology). The goal of these meetings could be to identify gaps in medical mycology training, accessibility to different antifungals, and access to diagnostics. These groups can provide training events and formulate regionally appropriate guidelines [41].

There is also a dearth of research on the level of not only physician awareness but also public awareness regarding IFIs in Middle Eastern countries. A national representative online survey in the US showed the level of awareness of IFIs among the general population was low, and it can be suspected that this would be similar in the Middle East. Therefore, educational programs on awareness of fungal infection among critical care or immunosuppressed patients and their caregivers or family members are required to raise public awareness of IFIs [63] (Figure 2).

## Conclusions

In conclusion, although information on epidemiology and IFI treatment management has become more streamlined in Middle Eastern countries, there exists a significant gap in standardizing IFI management practice in the region. Limited information on antifungal resistance and accessibility to advanced diagnostic tests are the key concerns. Moreover, the limited availability of biomarkers and lack of updated information on fungal infections create hurdles in practicing proper TDM. An MDT approach involving all the necessary stakeholders, including clinical pharmacologists, specialists, and pathologists, is requisite for better patient outcomes. In addition, local practice guidelines should be developed. Lastly, clinicians and laboratory specialists should be educated in medical mycology to improve overall IFI management.
